# The genetic and developmental enigma of rhizomes: crucial traits with limited understanding

**DOI:** 10.1007/s00122-026-05229-2

**Published:** 2026-05-07

**Authors:** Hongfei Chen, Jenn M. Coughlan

**Affiliations:** Department of Ecology and Evolutionary Biology, Yale University, New Haven, CT 06520 USA

## Abstract

Rhizomes, horizontal underground stems, play fundamental roles in plant persistence and perennial growth by enabling clonal propagation, resource storage, and stress resilience. Despite their ecological and agronomic importance across plant lineages, the genetic and developmental regulation of rhizomes remains poorly characterized. Here, we synthesize findings from in vitro induction studies, in vivo developmental analyses, quantitative trait loci (QTL) mapping, comparative transcriptomics, and limited functional studies to evaluate current knowledge and highlight outstanding questions in rhizome biology. Results from both in vitro and whole-plant studies show that phytohormones, particularly auxin, cytokinin, and gibberellin, are central regulators of rhizome initiation and growth, with effects mediated in a context-dependent manner through interactions with environmental and developmental cues. Across rhizomatous species, traits such as rhizome initiation, branching, and elongation are often polygenic, although comparatively simpler genetic architectures associated with repeated rhizome evolution have been documented in emerging model systems like *Mimulus*. Transcriptomic analyses further highlight hormone signaling, stress response, and carbohydrate metabolism pathways as key regulatory components. However, few genes have been functionally validated, underscoring the need for tractable systems for genetic dissection. Perennial *Mimulus* species are proposed as promising models for rhizome research due to their experimental accessibility, ecological relevance, and established genomic resources. Integrated approaches leveraging fine-mapping, near-isogenic lines, multi-omics, and gene editing are poised to accelerate discovery of causal loci and regulatory networks underlying rhizome development, thereby clarifying the genetic and developmental bases of rhizome traits underlying their repeated evolution, with broader implications for perenniality, environmental responses, and crop improvement.

**Supplementary Information:**

The online version contains supplementary material available at 10.1007/s00122-026-05229-2.

## Introduction

**Fig. 1:**
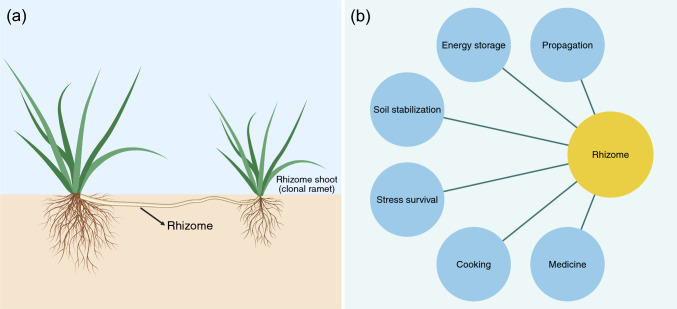
Structural and functional significance of rhizomes in plants. **a** Illustration of clonal propagation via rhizomes in a typical grass species, highlighting the production of a rhizome shoot (clonal ramet) emerging from a rhizome node and establishing an independent shoot–root module. **b** Key ecological and anthropogenic functions of rhizomes, including roles in propagation, energy storage, stress survival, and human use

Across diverse angiosperms, organ specialization has fundamental implications for both plants and people. In plants, such specialized organs have allowed for ecological expansion to novel habitats and can provide important services to the ecosystems in which they reside. Specialized organs are also often central to agricultural development, and much breeding effort focusses on the production of specialized organs in vegetable and medicinal crops. One particularly important specialized organ is rhizomes, which are modified stems that develop from axillary buds (Gizmawy et al. 1985) and grow horizontally underground (Fig. 1a) (Guo et al. 2021). Rhizomes hold substantial biological, ecological, and economic importance (Fig. 1b). Biologically, rhizome propagation is a common mode of asexual reproduction for perennial plants due to the rhizome’s capacity to generate new rhizomes, shoots, and roots (Dong and Pierdominici 1995; Duncan 1935; Huang et al. 2022). Many plants, such as bamboo (Kotangale et al. 2025; Uchimura 1980) and sheepgrass (Yang et al. 1995), rely primarily on rhizomes for natural propagation. On the other hand, the propagation through rhizomes can contribute to the weedy nature of certain plant species, such as Japanese knotweed and mugwort (Weston et al. 2005). Rhizomes are ecologically important traits. They function as storage organs for nutrients and energy that sustain many plants through winter dormancy, particularly in perennial grasses (Chung and Kim 2012; Dohleman et al. 2012; Mitros et al. 2020). This physiological reserve underpins whole-plant persistence of perennial herbs under a broad range of environmental stresses, including drought and cold, and enables regrowth following stress release (Guo et al. 2021; Ma et al. 2020; Tejera‐Nieves and Walker 2023). Consequently, compared to annual crops, perennial counterparts have some competitive advantages, including longer growing seasons and deeper root systems that tap water and nutrients at greater depths (Fan et al. 2020). Rhizomes can also provide important ecosystem services, such as reducing soil erosion (Balasuriya et al. 2018; Gyssels et al. 2005; Xue et al. 2016), and are economically important plant organs due to their nutritional and medicinal properties. For instance, the rhizomes of ginger, turmeric, and lotus are frequently utilized in various culinary applications, owing to their unique flavors and nutritional benefits. Furthermore, the rhizomes of medicinal plants such as *Salvia miltiorrhiza* (known as Danshen in Chinese) (Chong et al. 2019), *Panax ginseng* (Zhang et al. 2023), and *Atractylodes macrocephala* Koidz (known as Baizhu in Chinese) (Yang et al. 2021) are widely used in the treatment of various diseases, especially in Eastern Asian countries. Therefore, the identification of genes responsible for rhizome initiation and development is of utmost importance for crop breeding purposes, as rhizomes are integral to perenniality (Fan et al. 2020).

Although both arise from axillary buds on shoots, rhizomes are distinguished from their aboveground counterparts, stolons, or tillers, by their subterranean growth form. In grasses such as *Sorghum halepense*, axillary buds on seedling shoots undergo two orientation reversals, resulting in a positional gradient along the shoot, in which the lowermost buds oriented downward developed into rhizomes, whereas the more acropetally oriented buds developed into tillers (aerial, non-rhizomatous shoots) (Gizmawy et al. 1985). Similar bud reorientation and rhizome initiation were also observed in *Agropyron repens* (McIntyre 1967). The bud reversals were found to be driven by variations in radial growth rates between the internodes immediately above and below the bud, as well as by different cell division rates between the abaxial and adaxial sides of the bud (Gizmawy et al. 1985). Axillary buds on rhizomes can further either form new rhizomes or aerial shoots (Fisher 1972; Yoshida et al. 2016). Thus, although these specialized shoots both arise from axillary meristems and exhibit many developmental similarities (Guo et al. 2021), rhizomes are developmentally distinct from stolons, and this distinction arises early in their initiation.

While much is known about the developmental context of rhizome formation, particularly when and where rhizomes arise, a knowledge of how rhizome identity is genetically specified, regulated, and maintained is still in its infancy. Consequently, a broader synthesis regarding the regulation, molecular basis, and evolution of rhizomes is lacking. This knowledge gap largely stems from the absence of rhizomes in well-established model plant systems, such as *Arabidopsis* and tomato (Guo et al. 2021), but a collective body of recent research from rhizomatous grasses and other perennial taxa, including lotus, *Chrysanthemum*, and *Mimulus*, is providing important insights into the molecular genetic and developmental basis of these important organs. This review aims to synthesize current research on rhizome development, underlying molecular mechanisms, and their evolutionary significance, and to highlight promising directions for future studies in rhizome biology.

## Regulation factors of rhizome formation and growth

Rhizome formation and growth are shaped jointly by genetic regulators, phytohormonal pathways, and environmental cues. In vitro induction systems have clarified many of the hormonal requirements underlying rhizome initiation and growth, whereas in vivo studies reveal how these hormonal effects operate within whole-plant physiological and environmental contexts. Consistent with broader principles of plant developmental regulation, accumulating in vivo and *in planta* evidence indicates that phytohormones function not as autonomous triggers, but as integrative mediators that translate environmental cues into rhizome developmental outcomes.

Insights from in vivo and *in planta* studies across diverse rhizomatous species reveal that rhizome initiation and subsequent outgrowth are strongly conditioned by photoperiod, temperature, and seasonal cues, which in turn reshape endogenous hormone balances rather than acting through constitutive developmental programs. For example, in lotus (*Nelumbo nucifera*), rhizome enlargement occurs *in planta* under short-day photoperiods, and is accompanied by coordinated changes in auxin (Aux), gibberellic acid (GA), and abscisic acid (ABA) signaling, as shown by physiological hormone measurements and transcriptomic profiling of field-grown plants (Li et al. 2008; Masuda et al. 2006; Yang et al. 2015). Similar environment-dependent hormonal regulation has been reported in perennial grasses and rhizomatous crops, where drought stress modulates GA-dependent control of rhizome outgrowth (Zhang et al. 2022) and is associated with shifts in Aux, cytokinin (CK), and ABA pathways in intact plants, thereby influencing rhizome initiation, elongation, and storage-related carbohydrate metabolism (Ma et al. 2020). Together, these studies support a model in which phytohormones function primarily as integrative mediators that translate environmental information into developmental outcomes in vivo, rather than as isolated switches identified only under artificial induction conditions.

**Fig. 2:**
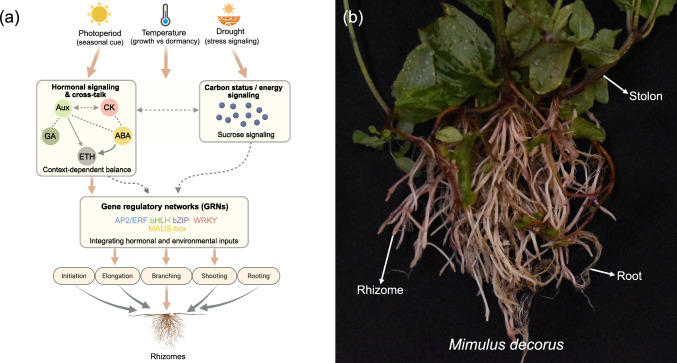
Conceptual framework of the molecular and morphological factors underlying rhizome development. **a** Conceptual model illustrating how rhizome development may be modulated based on current evidence. Dashed lines indicate an association/coupling (albeit with unresolved directionality). Bidirectional dashed arrows denote reciprocal interactions/feedbacks, whereas single dashed arrows indicate one-way influence suggested by available evidence without asserting strict hierarchy. Single solid arrows indicate directional effects supported by more direct experimental evidence. Large brown arrows represent the dominant “information flow” from environmental inputs through integrated physiological/hormonal states into GRNs and then into rhizome developmental outputs. Relationships are synthesized primarily from: Shimasaki and Uemoto 1991; Shimasaki 1993; Fukai et al. 2000; Roy and Banerjee 2002; Almeida et al. 2005; Kapoor and Rao 2006; Zahid et al. 2021; Ran et al. 2023. Aux, auxin; CK, cytokinin; GA, gibberellin; ETH, ethylene; ABA, abscisic acid. **b** Photograph of *Mimulus decorus* showing clear morphological differentiation between rhizomes (horizontal underground stems), stolons (horizontal aboveground stems), and roots

Guided by this integrated perspective, the following sections contrast insights from in vitro induction systems with evidence from in vivo studies, highlighting how hormonal requirements identified under controlled conditions are modulated by environmental context in intact plants (Table 1; Fig. 2a). At present, these relationships are best viewed as a conceptual synthesis based on available evidence rather than as a fully resolved molecular mechanism.

**Table 1:** Qualitative summary of phytohormonal and environmental influences on rhizome development

	Initiation	Elongation	Branching	Shooting	Rooting
Auxin	**↑/↑↓**	**↑/↑**	**↑/* − ***	**↑↓/ − **	**↑↓/* − ***
Cytokinin	**↑/↑**	**↑/↑**	**↓/* − ***	**↑/ − **	**↑↓/* − ***
Gibberellin	**↑/↑**	**↑/↑**	*** − */* − ***	**↑↓/↓**	**↓/* − ***
Ethylene	**↑/↑**	**↑/↑**	**↑/↑**	**↑↓/ − **	*** − */ − **
Jasmonic acid	**↑/ − **	*** − */↑**	*** − */* − ***	*** − */* − ***	*** − */* − ***
Abscisic acid	**↑/↑↓**	*** − */↑↓**	*** − */* − ***	*** − */* − ***	*** − */* − ***
Sucrose	**↑/* − ***	**↑/* − ***	*** − */* − ***	**↑/* − ***	**↑/* − ***
Nitrogen	*** − */↓**	*** − */ − **	*** − */ − **	*** − */↑**	*** − */ − **
Photoperiod	**↑/↑↓**	*** − */↑**	*** − */↑↓**	*** − */↑**	*** − */* − ***
Temperature	**↑/↑↓**	*** − */↓**	*** − */↓↑**	**↑/↓**	*** − */* − ***
Drought	**↑/↑↓**	*** − */↑↓**	*** − */* − ***	**↓/↑↓**	*** − */* − ***

Symbols indicate general trends synthesized across studies: ↑ promoting; ↓ inhibiting; ↑↓ context-dependent; − data not available. Effects from controlled in vitro systems and whole-plant in vivo studies are shown together in each cell as paired symbols (in vitro / in vivo). For environmental factors, photoperiod and temperature summarize responses to changes in environmental regimes (e.g., LD vs SD; low vs high temperature), such that effects can be promoting or inhibitory depending on context. Effects represent qualitative patterns rather than quantitative dose–response relationships. Actual outcomes vary with hormone concentration, stress intensity, species, developmental stage, tissue context, and experimental system. Detailed experimental conditions corresponding to each reported effect are provided in Supplementary Table S1

### Insights from in vitro studies

Many environmental and phytohormone cues influence aspects of rhizome development, both individually and in tandem. Here, we review evidence for the role of hormonal and abiotic cues in several aspects of rhizome development using in vitro studies.

#### Regulation factors of rhizome formation

CK and Aux are central regulators of rhizome initiation. CK alone can trigger rhizome formation in *Cyperus serotinus* (Omokawa et al. 1992) and in several Zingiberaceae species, including *Curcuma aromatica* (Nayak 2000) and *Zingiber officinale* (Abbas et al. 2014), whereas in orchids, Aux alone is sufficient and CK exerts little influence (Paek and Yeung 1991; Shimasaki and Uemoto 1991). In most systems, a finely balanced CK–Aux ratio is required for successful rhizome induction (Kapoor and Rao 2006; Roy and Banerjee 2002; Shimasaki and Uemoto 1991; Zahid et al. 2021). Additional hormones, including GA (Escalante and Langille 1995; Kapoor and Rao 2006), ABA (Kim et al. 2022), jasmonic acid (JA) (Rayirath et al. 2011), and ethylene (ETH) (Rayirath et al. 2011; Shimasaki 1993) act either downstream of or in concert with CK and Aux to modulate this developmental switch. Environmental conditions also influence rhizome formation, with high sucrose concentrations (Fan et al. 2022; Gezahegn et al. 2024; Kapoor and Rao 2006; Labrooy et al. 2020; Zahid et al. 2021), favorable photoperiod (Nayak 2000; Rout et al. 2001; Yoshida et al. 2016), optimal temperature (Aleixo and Valio 1976), and mild drought stress (Almeida et al. 2005) promoting induction.

#### Regulation factors of rhizome elongation and branching

Many phytohormones and environmental cues can stimulate rhizome elongation; however, the effects of specific phytohormones are not always consistent. For example, CK and Aux both promote rhizome elongation in *Gastrodia elata* (Hsieh et al. 2022). GA similarly enhances elongation in *Caulerpa prolifera* (Jacobs and Davis 1983) and *Solanum tuberosum* (Escalante and Langille 1998), whereas ETH, while generally inhibitory (Vaseva et al. 2018), can stimulate elongation in *Rheum rhabarbarum* (Rayirath et al. 2011) under certain conditions. Higher sucrose concentrations also promote rhizome elongation in *Acorus calamus*, *Bambusa bambos,* and *Oryza longistaminata* (Fan et al. 2022; Kapoor and Rao 2006; Subramani et al. 2014).

Rhizome branching is also influenced by phytohormones. For example, Aux typically stimulates rhizome branching in *Cymbidium aloifolium* (Nayak et al. 1998), *Cymbidium forrestii* (Paek and Yeung 1991), and *Geodorum densiflorum* (Roy and Banerjee 2002), whereas CK tends to inhibit it in orchid species such as *Cymbidium kanran* (Shimasaki 1995) and *C. forrestii* (Paek and Yeung 1991). Depending on concentration and species, CK can either counteract (Roy and Banerjee 2002) or enhance Aux-driven branching (Fukai et al. 2000). Beyond Aux and CK, ETH promotes branching in *R. rhabarbarum* (Rayirath et al. 2011) and *C. kanran* (Shimasaki 1995).

#### Regulation factors of rhizome shooting and rooting

Lastly, much evidence supports the role of phytohormones in the formation of rhizome shoots and rooting phenotypes. As with other phenotypes, CK strongly induces rhizome shoot formation across *Alstroemeria* (Khaleghi et al. 2008; Shahriari et al. 2012), *A. calamus* (Tikendra et al. 2022), *C. kanran* (Shimasaki 1995; Shimasaki and Uemoto 1990), *C. aloifolium* (Nayak et al. 1998), *G. densiflorum* (Roy and Banerjee 2002), and *Kaempferia galang* (Vincent et al. 1992). Aux similarly promotes shoot initiation in *Alstroemeria* (Hamidoghli et al. 2007; Khaleghi et al. 2008; Shahriari et al. 2012) and *G. densiflorum* (Roy and Banerjee 2002) but inhibits it in *A. calamus* (Tikendra et al. 2022). GA enhances rhizome shooting in *Zantedeschia* (Kozłowska et al. 2007) and *R. rhabarbarum* (Rayirath et al. 2009) yet suppresses it in *A. repens* (Rogan and Smith 1976). ETH likewise inhibits rhizome shoot development in *C. kanran* (Ogura-Tsujita and Okubo 2006; Shimasaki 1995) and *Kohleria eriantha* (Almeida et al. 2005), while promoting it in *Z. officinale* (Furutani et al. 1985). Environmental cues, including sucrose (Paek and Yeung 1991; Zahid et al. 2021), temperature (Leakey et al. 1978), and drought (Rice et al. 1996), also exert strong, often context-dependent effects on rhizome shoot induction.

CK promotes rhizome rooting in *Valeriana jatamansi* (Nazir et al. 2022) but inhibits it in *Alstroemeria* (Gabryszewska and Hempel 1985; Kristiansen et al. 1999), *G. densiflorum* (Roy and Banerjee 2002), and *Ruppia maritima* (Koch and Durako 1991). Similarly, Aux promotes rooting in *Alstroemeria* (Hamidoghli et al. 2007; Khaleghi et al. 2008; Kristiansen et al. 1999), *Podophyllum hexandrum* (Nadeem et al. 2000), *Posidonia oceanica* (Balestri and Lardicci 2006), *Scopolia parviflora* (Kang et al. 2004), and *V. jatamansi* (Nazir et al. 2022) but suppresses it in *Ruppia maritima* (Koch and Durako 1991). GA inhibits root initiation in *Convolvulus sepium* (Wells and Riopel 1972), whereas sucrose enhances rooting in *Alstroemeria* (Gabryszewska 1996) and *Z. officinale* (Zahid et al. 2021).

#### Synthesis and future directions for in vitro research

Collectively, in vitro studies reveal that rhizome formation and growth are regulated primarily by an Aux–CK axis, defined here as the dominant interplay between Aux and CK signaling, with GA, ETH, and sucrose modulating its outcome under specific environmental contexts. However, because most experiments expose isolated tissues to exogenous treatments under simplified conditions, they remain largely descriptive and offer limited insight into how these signals are integrated within intact plants. Future in vitro work should therefore move beyond simple induction assays toward more mechanistic designs, employing more precisely parameterized hormonal and environmental manipulations together with molecular and cellular readouts. In particular, in vitro systems that allow fine-scale spatial and temporal control of signaling inputs, for example through engineered culture platforms, may better capture key aspects of rhizome polarity and early developmental transitions. Such approaches will more effectively resolve causal regulatory relationships and generate testable hypotheses about conserved versus context-dependent regulatory modules for evaluation in vivo.

### Insights from in vivo studies

#### Meristem fate determination and rhizome initiation

Although much remains unknown about the specific cell types that give rise to rhizomes, new work in *O. longistaminata* using single-cell and spatial transcriptomic analyses is providing novel insights. This work identified meristematic initiation cells within a sunken parenchyma zone at the internode base as the starting point for rhizome initiation (Lian et al. 2024). Furthermore, trajectory analysis revealed that rhizomes originate de novo through cell fate reprogramming (Lian et al. 2024). Complementary morphological evidence indicates that axillary bud shape predicts developmental fate: dome-shaped buds penetrate the leaf sheath to produce rhizomes, whereas flat buds remain enclosed and develop into tillers (Wang et al. 2024b).

#### Nutritional and environmental control

Environmental and nutritional factors exert strong, species-specific influences on rhizome initiation and elongation across clonal plants. A series of studies in *A. repens* established that increased water availability, low temperature, reduced nitrogen, and long-day photoperiods promote rhizome formation, while conditions such as drought, high temperature, and short-day photoperiods favor tillering (McIntyre 1964, 1967, 2001; McIntyre and Cessna 1998). However, these patterns are not consistent across species. In *Lotus corniculatus*, for instance, rhizome formation is enhanced under short-day autumn conditions (Kallenbach et al. 2001), whereas exposure to cold suppresses rhizome formation in *Poa pratensis* (Moser et al. 1968). Temperature and photoperiod jointly determine elongation capacity. In *Poa pratensis*, long-day and high-temperature conditions promote rhizome elongation (Aamlid 1992; Moser et al. 1968) and in *O. longistaminata*, higher temperatures similarly lead to substantially longer rhizomes (Wang et al. 2024a). Additionally, continuous light accelerates elongation in *C. prolifera* (Chen 1971), and long-day conditions similarly enhance rhizome extension in *Zantedeschia* (Anderson 1970). Drought typically reduces rhizome initiation and elongation in mesic species such as *Festuca arundinacea* (Ma et al. 2020), *Leymus chinensis* (Wang et al. 2019), *Carex lasiocarpa* (Yuan et al. 2017), and *Chrysanthemum morifolium* (Zhang et al. 2022), but can instead enhance clonal spread in stress-adapted taxa, including *Triglochin buchenaui* (Tabot and Adams 2012) and *Leymus secalinus* (Zheng et al. 2021).

The environmental regulation of rhizome branching and shoot emergence is similarly complex. Photoperiodic sensitivity varies markedly across species: in *N. nucifera*, rhizome branching increases under long, warm days (Masuda et al. 2006), whereas in *Alstroemeria*, long days suppress branching and cooler temperatures promote branch formation (Vonk Noordegraaf 1981). High nitrogen supply, long days, and elevated temperature stimulate rhizome-derived shoot formation in *Cyperus esculentus* (Garg et al. 1967), whereas *Phragmites australis* exhibits a drought-dependent response, with shoot production increasing after 90 days but becoming suppressed after 120 days (Mingyang et al. 2022).

#### Hormonal cross-talk and environmental integration

In *P. pratensis*, the rhizome-abundant ecotype exhibits elevated CK and a low Auxin/CK ratio, a hormonal balance associated with enhanced rhizome formation (Ran et al. 2023), and exogenous GA₃ and IAA similarly promote rhizome formation and upward turning in *L. secalinus* (Li et al. 2022a). Stage-resolved hormonal profiling in *N. nucifera* further shows that CK peaks at the early formation stage, whereas GA, IAA, and SA increase during swelling and ABA accumulates predominantly in late swelling (Li et al. 2008), indicating a shift from early cytokinin enrichment to GA/IAA-associated expansion and ABA-associated maturation. This stage-specific progression highlights that rhizome development relies on the coordinated action of multiple hormone classes. In line with this broader pattern, GA stimulates rhizome formation in *C. morifolium* (Zhang et al. 2022) and promotes elongation in *F. arundinacea* (Ma and Huang 2016; Ma et al. 2016), but suppresses rhizome-derived shoot formation in *C. esculentus* (Garg et al. 1967). ETH likewise enhances rhizome elongation and branching in *S. tuberosum* (Langille 1972). Hormone–environment interactions further modify these processes. In *K. eriantha* (Gesneriaceae), drought or low water availability increases ABA levels, which stimulate ETH production, suppress aerial bud growth, and promote rhizome differentiation (Almeida et al. 2005). By contrast, in *F. arundinacea*, drought suppresses rhizome initiation and growth and is accompanied by increased ABA and soluble sugar accumulation, whereas post-drought recovery involves rises in IAA, CK, and GA that reflect a sequential hormonal rebalancing coordinated with energy metabolism (Ma et al. 2020).

#### An ecological synthesis of contrasting responses

Across rhizomatous species, environmental effects on rhizome initiation and growth frequently appear contradictory when considered in isolation (Table 1). From an ecological perspective, however, these divergent outcomes largely reflect differences in the primary functions that rhizomes serve across habitats, rather than simple inconsistency in developmental regulation. Temperature responses illustrate this clearly. In species occupying environments where seasonal cold represents a major survival challenge, rhizomes function primarily as overwintering and belowground storage organs, and exposure to low temperature or seasonal cooling can favor rhizome initiation or increased belowground investment, as demonstrated in the cold-tolerant, overwintering perennial *A.* repens (McIntyre 1967; McIntyre and Cessna 1998). By contrast, in species inhabiting environments where rhizomes mainly support active vegetative growth and spatial spread during favorable seasons, low temperature acts primarily as a constraint on overall growth and consequently suppresses rhizome development, as observed in the cool-season perennial grass *P. pratensis* (Moser et al. 1968). Within this same ecological framework, variation in temperature predominantly shapes the extent to which rhizomes contribute to clonal spread during the growing season, as reflected by enhanced rhizome elongation under warmer conditions in the tropical perennial *O. longistaminata* (Wang et al. 2024a). Responses to drought show a similarly predictable pattern when interpreted through differences in habitat specialization and stress tolerance. In drought-adapted or xeric species, mild or episodic water limitation can enhance rhizome formation or clonal spread, consistent with the use of underground stems as persistence or escape structures, as reported in taxa such as *T. buchenaui* and *L. secalinus* (Tabot and Adams 2012; Zheng et al. 2021). In contrast, in mesic or moist-adapted species, drought more commonly suppresses rhizome initiation and elongation, reflecting developmental limitations under water deficit, as observed in *F. arundinacea*, *L. chinensis*, *C. lasiocarpa*, and *C. morifolium* (Ma et al. 2020; Wang et al. 2019; Yuan et al. 2017; Zhang et al. 2022). Rhizome branching and shoot emergence also show pronounced species- and context-specific variability, reflecting sensitivity to developmental stage, resource availability, and the timing of environmental cues. Together, these patterns indicate that rhizome development is a context-dependent outcome shaped by ecological strategy and life-history adaptation. Recognizing this ecological conditionality provides a clearer framework for interpreting comparative results and for guiding future studies.

#### Synthesis and future directions for in vivo research

Compared with in vitro systems, in vivo studies reveal more species-specific and context-dependent outcomes (Table 1), reflecting the combined action of multiple interacting factors within an integrated physiological framework. Nevertheless, such work has yielded broader, system-level insights into how hormonal cross-talk interfaces with developmental polarity, environmental cues, and carbon allocation to shape rhizome development in whole plants. Despite these advances, most in vivo studies rely on endpoint sampling under specific environmental or developmental conditions, restricting our understanding of the dynamic and spatial regulation of rhizome development, and differences in developmental staging, environmental treatments, and sampled tissue types across studies further complicate direct comparisons among species. To address these constraints, recent methodological advances now permit higher resolution in vivo analyses: single-cell and spatial transcriptomic approaches can identify cell-type-specific transcriptional programs associated with the specification of axillary meristem fate toward rhizome development within intact tissues; live hormone reporters (e.g., DR5, TCSn, and DII-VENUS), when combined with confocal or light-sheet microscopy, enable direct visualization of dynamic hormone distributions and their spatial relationship to growth directionality and tissue differentiation; and chromatin-level assays such as ATAC-seq or CUT&RUN applied to fate-specified buds or rhizome meristems provide a tractable strategy for identifying *cis*-regulatory elements and transcription factor binding events underlying rhizome-specific developmental programs. Together, these emerging approaches offer a concrete framework for linking hormonal, environmental, and regulatory dynamics to rhizome development in vivo.

## The molecular mechanisms underlying rhizome initiation and growth

### The genetics of rhizome development

The genetic basis of rhizome initiation and growth has been primarily investigated in economically important crops such as rice (Fan et al. 2020; Hu et al. 2003; Li et al. 2022b; Wang et al. 2024b; Yoshida et al. 2016), sorghum (Kong et al. 2015, 2022; Paterson et al. 1995), and lotus (Huang et al. 2021). These studies have focused on two major developmental aspects: rhizome initiation and subsequent growth traits contributing to rhizome abundance (e.g., rhizome number, branching degree, or spread perimeter). In crops, the presence or absence of rhizomes is generally governed by a polygenic architecture. For instance, Kong et al. (2015) identified at least four loci underlying rhizomatousness between *Sorghum bicolor* and *Sorghum propinquum*, while Hu et al. (2003) reported two dominant-complementary loci, *Rhz2* and *Rhz3*, that regulate rhizome initiation in a cross between cultivated rice (*Oryza sativa*) and wild rice (*O. longistaminata*). Using higher-density markers, Li et al. (2022b) further identified 13 loci associated with rhizome presence in the same species pair and showed that three or more alleles from *O. longistaminata* were necessary for rhizome formation in a recombinant inbred line (RIL) population. Similarly, traits contributing to rhizome abundance have been shown to be polygenically controlled in crops, with key traits including rhizome number (Hu et al. 2003; Huang et al. 2021; Kong et al. 2015; Li et al. 2022b), branching degree (Hu et al. 2003; Li et al. 2022b), and spread perimeter (Larson et al. 2014). However, recent research in *Mimulus* suggests that both rhizome initiation and growth traits underlying abundance (e.g., number, branching, length, width) follow a comparatively simple genetic architecture in crosses between multiple high-altitude perennials and a shared low-altitude perennial (Chen et al. 2025; Coughlan et al. 2021). This contrast may reflect fundamental differences in evolutionary trajectories and ecological strategies across lineages. In crops, rhizomes are often associated with conserved and multifunctional roles such as vegetative reproduction, nutrient storage, and perennial growth, as documented in perennial rice, rhizomatous sorghum, *Miscanthus*, and switchgrass, in which rhizomes function as long-term belowground biomass reserves supporting seasonal regrowth and persistence (Dohleman et al. 2012; Mitros et al. 2020; Paterson et al. 2020; Shanmugam et al. 2025; Silva et al. 2024; Tejera‐Nieves and Walker 2023; Tong et al. 2023), roles that are likely underpinned by more layered genetic regulation. By contrast, in *Mimulus*, rhizomes appear to serve primarily as an adaptive trait enabling perenniality in cold, high-elevation environments where vegetative persistence is advantageous, a pattern reflected in both the frequent co-occurrence of rhizomatous growth and perennial life-history strategies in high-altitude taxa and a comparatively less complex genetic architecture underlying rhizome initiation and growth (Chen et al. 2025; Coughlan et al. 2021). Furthermore, although rhizome initiation and subsequent growth traits (e.g., axillary bud fate specification versus rhizome elongation and girth enlargement) are often treated as distinct developmental phases in anatomical and physiological studies (Guo et al. 2021; Yang et al. 2015; Yoshida et al. 2016), QTL mapping often reveals overlapping loci controlling both processes, suggesting shared regulatory mechanisms. For example, *Rhz2* and *Rhz3*, which control rhizome presence in rice, were also found to influence traits such as branching and internode length (Hu et al. 2003), and all five QTLs associated with rhizome number in sorghum were reported to overlap with regions controlling rhizome presence (Kong et al. 2015), collectively pointing to the interpretation that pleiotropic or tightly linked loci can influence both rhizome initiation and subsequent elaboration.

Rhizomes develop from axillary buds, and accumulating evidence suggests a close genetic relationship between axillary bud regulation and rhizome development. For example, Paterson et al. (1995) demonstrated that one QTL influenced the number of available axillary buds, while several others determined the fate of these buds, that is, whether they differentiate into rhizomes or tillers, in crosses between wild and cultivated *S. bicolor*. Similarly, Kong et al. (2015) found that some QTLs associated with rhizome occurrence in *S. bicolor* × *S. propinquum* overlapped with QTLs controlling tiller number and axillary branching, suggesting that rhizomatousness and aerial shoot branching may share common developmental pathways. This pattern was further supported by Kong et al. (2022), who identified overlapping QTLs for rhizomatousness, tillering, and branching in *S. bicolor* × *S. halepense* crosses. In addition to these shared regulatory axes, Hu et al. (2003) observed that QTLs controlling rhizome initiation and growth traits underlying abundance in *O. longistaminata* largely corresponded to homologous regions in the *S. propinquum* genome, indicating that certain loci involved in rhizome development may be conserved across Poaceae lineages. Although causal genes have yet to be fully identified, these findings collectively provide important insights into the genetic mechanisms underlying rhizome development and lay the groundwork for future gene-level discovery through integrative genetic and genomic approaches.

### Gene regulation networks of rhizomes based on multiple expression data

Despite the lack of identified causal genes directly responsible for rhizome initiation and proliferation in natural plant populations, extensive transcriptomic investigations have been conducted across a wide array of rhizomatous species to uncover candidate genes potentially involved in rhizome development. These high-throughput gene expression studies span economically and ecologically important species such as *O. longistaminata* (He et al. 2014; Hu et al. 2011; Wang et al. 2024a; Zhang et al. 2015), *S. halepense* and *S. propinquum* (Jang et al. 2006; Zhang et al. 2014), *N. nucifera* (Cheng et al. 2013; Ming et al. 2013; Yang et al. 2015), *A. lancea* (Huang et al. 2016), *Miscanthus lutarioriparius* (Hu et al. 2017), *Trifolium ambiguum* (Meng et al. 2021), *L. chinensis* (Gao et al. 2023), as well as *Z. officinale* and *Curcuma. longa* (Koo et al. 2013). A convergent finding across these studies is the recurrent involvement of phytohormone-related genes in rhizome development, particularly those involved in Aux, CK, ETH, GA, and ABA signaling pathways. For example, transcriptomic analyses comparing stolon tips (at the rhizome induction stage) with other stages of rhizome development (e.g., swelling stages) in *N. nucifera* revealed elevated expression of auxin- and ethylene-responsive genes, suggesting a role for these pathways in rhizome initiation (Cheng et al. 2013). Most transcriptomic studies, however, have focused on differential gene expression between rhizome tissues and non-rhizome tissues (e.g., leaves). In *O. longistaminata*, genes involved in Aux, GA, and ETH signaling showed higher expression in rhizomes than in leaves, roots, and stems (He et al. 2014), whereas in *A. lancea*, CK- and AUX-related genes were preferentially upregulated in rhizome tissues (Huang et al. 2016). Similar hormone-related expression patterns have also been described across the rhizomatous taxa listed above, reinforcing the central role of hormone-mediated regulatory networks in rhizome development across diverse lineages.

Moreover, many genes associated with biotic and abiotic stress responses, such as heat shock proteins, have been reported to be highly expressed in rhizome tissues across multiple species (He et al. 2014; Huang et al. 2016; Ming et al. 2013). In parallel, genes involved in energy metabolism, particularly those related to starch and sucrose pathways, also exhibit elevated expression in rhizomes (Cheng et al. 2013; Hu et al. 2017; Huang et al. 2016; Meng et al. 2021), consistent with the established roles of rhizomes in nutrient storage and overwintering. Recent ‘omics work on rhizomatous tissues also commonly reports elevated expression of lignin-biosynthetic genes, indicating that enhanced cell-wall reinforcement is another recurring feature of rhizome development (Ma et al. 2024; Meng et al. 2021; Yang et al. 2019; Cao et al. 2023). In addition, a suite of transcription factors, including members of the AP2/ERF, bHLH, bZIP, WRKY, and MADS-box families, are commonly expressed in rhizomatous tissues across diverse taxa (Fig. 2a) (Cheng et al. 2013; He et al. 2014; Hu et al. 2011; Huang et al. 2016; Koo et al. 2013; Meng et al. 2021; Ming et al. 2013; Ruan et al. 2021; Yang et al. 2015; Zhang et al. 2014). These transcription factors are broadly implicated in plant hormone signaling and environmental stress responses, reinforcing the view that rhizome development is tightly coordinated by integrated hormonal and stress-response regulatory networks.

### Genes functionally validated to influence rhizome development

To date, only a few genes have been functionally validated to influence rhizome development. One such gene is *BLADE-ON-PETIOLE* (*BOP*), a conserved regulator of proximal–distal leaf patterning (Hepworth et al. 2005; Toriba et al. 2019) rather than a rhizome-specific factor. In the rhizomes of *O. longistaminata*, high *BOP* activity maintained by miR156 suppresses leaf blade formation, yielding compact, sheath-dominated leaves; *bop* loss-of-function mutants instead produce ectopic blades on rhizome leaves, which reduces tip stiffness and impairs penetration through soil (Toriba et al. 2020). Thus, *BOP* primarily controls leaf identity, but this modulation of rhizome leaf morphology has important consequences for underground growth mechanics. Two additional genes, *CmRH56* and its downstream target *CmGA2ox6*, have been identified in *Chrysanthemum* (*C. morifolium*) (Zhang et al. 2022). *CmRH56*, a DEAD-box RNA helicase specifically expressed in the rhizome shoot apex, regulates rhizome outgrowth under drought stress: RNAi knockdown lines produce fewer rhizomes, whereas overexpression accelerates and increases rhizome formation. Mechanistically, *CmRH56* represses the GA-catabolic gene *CmGA2ox6*. Silencing *CmGA2ox6* leads to a marked increase in rhizome number, indicating that GA availability promotes rhizome outgrowth. Importantly, however, current evidence does not distinguish whether GA signaling specifically biases axillary buds toward a rhizome identity, or more generally enhances lateral bud outgrowth that yields both aerial and subterranean stems. Despite this uncertainty, these gene-based studies provide initial insights into the molecular mechanisms underlying rhizome development.

### Synthesis and future directions for genetic and regulatory studies of rhizomes

Although genetic mapping, expression profiling, and limited functional studies have begun to identify loci and pathways involved in rhizome initiation and growth, the mechanisms that specify rhizome identity and govern its subsequent growth remain poorly understood. In particular, it is still unclear how hormonal cues, environmental signals, and developmental regulators interact to direct an axillary bud toward a rhizome rather than an aerial shoot. Addressing these questions will require moving from correlation to causation through precise genetic dissection of the bud-to-rhizome transition and its downstream developmental programs. Recent advances in single-cell and spatial transcriptomics, chromatin accessibility profiling (e.g., ATAC-seq and CUT&RUN), and CRISPR-based gene perturbation now make it feasible to link candidate regulatory elements and gene networks to specific developmental outcomes in rhizome-forming systems. When integrated with fine-scale genetic mapping and near-isogenic line analyses, these approaches provide a tractable framework for identifying the core regulatory switches that initiate and sustain rhizome development.

## Evolutionary patterns and adaptive significance of rhizomatous growth

### Phylogenetic context and repeated evolution of rhizomatous growth

Rhizomatous growth occurs across a wide phylogenetic breadth within vascular plants, spanning multiple monocot and eudicot lineages adapted to aquatic, mesic, and seasonally stressful terrestrial environments (Guo et al. 2021). The absence of rhizomes in many closely related species, together with pronounced variation in rhizome characteristics and ecological function, is consistent with multiple independent evolutionary origins shaped by similar selective pressures (Chen et al. 2025; Hu et al. 2003; McDowell and Gang 2012). However, formal ancestral-state reconstructions remain limited for most groups, and the evolutionary lability of rhizomes in some genera may suggest the presence of rhizomes as an ancestral character with subsequent loss in some taxa (Tribble et al. 2021).

Across lineages, rhizomes are consistently associated with ecological contexts in which long-term persistence, stress buffering, and vegetative regeneration are favored (Guo et al. 2021; Liu et al. 2016). In cold or strongly seasonal environments, rhizomes function as protected belowground storage organs that enable overwinter survival and rapid regrowth (Guo et al. 2021; McDowell and Gang 2012). In drought-prone or disturbance-prone habitats, rhizomes are often associated with subterranean growth forms that may contribute to persistence during unfavorable periods, although alternative life-history strategies such as annuality or shifts in mating system (e.g., self-compatibility) can also be favored in these environments (Grossenbacher et al. 2015; Guo et al. 2021; McDowell and Gang 2012). These recurring functional roles point to rhizomes as a repeatedly favored adaptive solution for perennial life histories.

At the developmental level, rhizomes develop from axillary buds, and genetic mapping studies across grasses and other taxa repeatedly reveal partial overlap between loci controlling axillary bud number, branching, tillering, and rhizome formation (Guo et al. 2021; Hu et al. 2003; Kong et al. 2015; Paterson et al. 1995). Comparative transcriptomic studies further show recurrent involvement of hormone signaling pathways (Aux, CK, GA, ETH, and ABA), carbohydrate metabolism, and cell-wall reinforcement across distantly related rhizomatous species (Cheng et al. 2013; Guo et al. 2021; He et al. 2014; Huang et al. 2016; Meng et al. 2021). These pathways are broadly conserved regulators of shoot development, suggesting that rhizomes frequently evolve via quantitative or context-dependent redeployment of existing regulatory networks (Carroll 2008; Kopp 2009). Importantly, repeated emergence of rhizomatous growth does not imply repeated evolution of identical genetic mechanisms. Instead, available evidence is consistent with a model in which rhizomes arise through the co-option of conserved developmental and genetic modules, rather than through the origin of rhizome-specific genes, with different lineages potentially recruiting overlapping but non-identical components of these shared networks (Chen et al. 2025).

Together, these observations support a modular co-option framework in which rhizomes represent a convergent architectural outcome assembled from shared developmental components. This framework reconciles the repeated evolution of rhizomatous growth with the absence of clear candidates for universally conserved rhizome-specific genes and yields testable predictions regarding where and how convergence is expected to occur across lineages. Specifically, independent rhizomatous lineages may exhibit convergence at the level of regulatory networks or quantitative trait loci, rather than strict gene-level homology. Integrating phylogenetic comparative analyses with genetic mapping, functional genomics, and developmental studies in experimentally tractable systems will be essential for distinguishing shared evolutionary “hotspots” from lineage-specific solutions.

### Ecological roles and adaptive consequences of rhizomatous growth

At the ecological and functional level, rhizomes play pivotal roles in vascular plants by enhancing stress tolerance, facilitating vegetative spread, and shaping plant–environment interactions across diverse habitats. In monocots, an early evolutionary condition has been hypothesized to have been aquatic with a rhizomatous growth form, which enabled plants to anchor in shallow water while adjusting to fluctuations in water and sediment levels, thereby improving gas exchange efficiency (Howard et al. 2019). Some studies further propose that the rhizome represents a morphological precursor to the upright stem (McDowell and Gang 2012), and environmental factors such as temperature have likely served as key selective pressures shaping rhizome diversity (Howard et al. 2019). The evolutionary significance of rhizomes becomes particularly evident in the context of plant invasiveness, where rapid evolution is frequently observed. For instance, *S. halepense*, native to western Asia, exhibits a strong association between its rhizomatous growth and its acquired cold tolerance following introduction to North America (Paterson et al. 2020). Similarly, *Artemisia vulgaris*, a European native, has evolved extensive rhizome networks that contribute to its invasiveness across the Northeastern and Mid-Atlantic USA (Barney et al. 2009). Rhizomes confer adaptive advantages under extreme conditions such as drought and frost, primarily by serving as energy storage organs that support regrowth after stress or dormancy (McDowell and Gang 2012). Additionally, rhizomes enhance ecosystem engineering by improving soil stabilization, which may have contributed to the successful terrestrial radiation of early vascular plants (Xue et al. 2016). Together, these examples illustrate how rhizomes associated with evolutionary persistence contribute to plant invasiveness, enhanced stress tolerance, and ecosystem engineering across a wide range of environments.

### Synthesis and future directions for rhizome evolution

Although rhizomatous growth has evolved repeatedly across vascular plant lineages, the extent and level at which evolutionary convergence occurs remain unresolved. A central challenge for future work is to rigorously test whether independent origins of rhizomes reflect convergence at the level of individual genes, genomic regions, or higher-order developmental programs under similar ecological conditions. Addressing this question will require explicitly comparative, phylogenetically informed study designs, in which multiple independently evolved rhizomatous systems are analyzed in parallel. Genetic mapping, functional assays, and regulatory profiling in these systems should be deployed not to further dissect rhizome formation per se, but to test whether similar evolutionary “hotspots” are repeatedly recruited or whether distinct lineage-specific solutions predominate. Integrating such comparative analyses with emerging technologies, including single-cell and spatial transcriptomics, chromatin accessibility profiling, and CRISPR-based gene perturbation applied across systems, will allow direct evaluation of evolutionary convergence versus divergence, moving the field beyond descriptive patterns toward testable evolutionary inference.

## *Mimulus* as a model system for rhizome biology

While extensive research has been conducted on rhizomes across diverse plant taxa, key mechanistic insights remain limited. Real progress on the questions and approaches outlined above will require a system that integrates natural variation in rhizome traits with strong genetic and genomic tractability, making the identification of an appropriate model organism essential for advancing rhizome research.

Several rhizome-forming systems have already delivered key breakthroughs, including genetically mapped loci and a limited set of genes with functional evidence affecting rhizome growth or morphology, and therefore provide indispensable foundations for the field. Notably, perennial rice and its wild rhizomatous relatives (*O. longistaminata* and derived introgression materials) offer an exceptional monocot framework in which rhizome development can be studied in the context of wild-cultivated contrasts (Hu et al. 2003; Li et al. 2022b), within a well-developed rice genomic framework. In addition to *O. longistaminata*, several other perennial wild rice species (e.g., *O. officinalis*, *O. australiensis*, and *O. rhizomatis*) exhibit rhizomatous growth, highlighting diversity within *Oryza* (Tong et al. 2023). A major strength of the *Oryza* system is the availability of reference genomes, dense molecular markers, and agronomically interpretable phenotypes, which together enable high-resolution QTL detection and comparative mapping (Paterson et al. 1995; Wing et al. 2018). However, rhizomes in this framework are largely restricted to perennial wild relatives, which often have long generation times (Cissé and Khouma 2016) and comparatively limited transformability or genome-editing efficiency (Table 2; Shimizu-Sato et al. 2020). In addition, introgression lines frequently carry large donor segments, such that rhizome presence can be coupled with linked, pleiotropic, or background-dependent traits, making iterative fine-mapping and clean causal validation challenging in practice (Hasan et al. 2015; Paterson et al. 1995). Rhizomatous sorghums (e.g., *S. halepense* and *S. propinquum*) have similarly been powerful for establishing the genetic architecture of rhizomatousness and for connecting rhizome traits to broader axillary bud and branching biology (Kong et al. 2015, 2022; Paterson et al. 1995). A notable advantage of this system lies in strong synteny and translational relevance within grasses, which supports cross-species inference and candidate prioritization (Paterson et al. 1995). Beyond grasses, horticultural rhizome crops such as lotus (*N. nucifera*) provide a complementary strength: rhizome development is anatomically conspicuous and agriculturally central, enabling precise developmental staging (e.g., initiation versus swelling) and targeted sampling of key transitions (Cheng et al. 2013; Li et al. 2008; Ming et al. 2013; Yang et al. 2015). This has made lotus an excellent system for associating hormonal dynamics and transcriptomic programs with stage-specific developmental processes. Nevertheless, both rhizomatous sorghums and horticultural rhizome crops remain comparatively slow for genetic dissection at scale, as life-cycle constraints and less routine transformation or genome editing restrict the ability to rapidly test causality across many candidate loci or regulatory elements (Azhakanandam and Zhang 2015; Cox et al. 2018; Deng et al. 2020; Namata et al. 2025; Zhou et al. 2022). Likewise, *Chrysanthemum* (*C. morifolium*) has yielded some of the clearest gene-level examples of regulators influencing rhizome outgrowth under stress (Zhang et al. 2022). Its strengths lie in well-developed horticultural manipulation, strong phenotypic signals under defined stress conditions, and direct applied relevance. However, *Chrysanthemum* also poses well-known challenges for mechanistic dissection, including high heterozygosity and/or polyploidy, background-dependent transgene performance, longer generation times, and reduced feasibility of producing large, clean segregating populations for high-resolution mapping and repeated validation (TeixeiradaSilva and Kulus 2014). More broadly, most existing model systems fall short of meeting these requirements, and classical genetic models such as *Arabidopsis thaliana*, *Solanum lycopersicum*, *Brachypodium distachyon, Setaria viridis*, and *Populus trichocarpa* do not produce rhizomes, fundamentally limiting their utility for studying rhizome initiation or underground stem development. However, these systems remain invaluable for elucidating conserved developmental and genetic modules (e.g., axillary meristem regulation, branching, and hormonal control) that are likely co-opted during rhizome evolution.

**Table 2:** Comparison of major plant model systems relevant to dissect rhizome biology

Model system	Rhizomes	Generation time	Genome size	Natural variation in rhizomes	Transformation efficiency
*Oryza sativa* (rice)	✕ in *O. sativa*; √ in wild relative	~ 4 months	~ 400 Mb	✕ (via *O. longistaminata* introgression)	√ *O. sativa*; √ *O. longistaminata*
*Sorghum halepense* / *S. propinquum*	√	~ 3–4 months	~ 730 Mb	√	✕
*Nelumbo nucifera*	√	~ 3–5 months	~ 929 Mb	√	✕
*Chrysanthemum morifolium*	√	~ 3–4 months	~ 9 Gb	√	✕
*Arabidopsis thaliana*	✕	~ 6 weeks	~ 135 Mb	✕	√
*Solanum lycopersicum* (tomato)	✕	~ 3 months	~ 900 Mb	✕	√
*Brachypodium distachyon*	✕	~ 8 weeks	~ 272 Mb	✕	√
*Setaria viridis*	✕	~ 6 weeks	~ 510 Mb	✕	√
*Populus trichocarpa* (Poplar)	✕	~ 6 months (early-flowering lines)	~ 480 Mb	✕	√
*Mimulus* (monkeyflowers)	√	~ 6 weeks	~ 430 Mb	√ extensive	√

Summary of key features of representative plant systems, including generation time, genome size, presence of rhizomes, natural variation in rhizome traits, and feasibility of genetic transformation, to facilitate comparative evaluation across systems. These systems include both rhizome-forming species and non-rhizomatous model organisms, the latter of which provide important genetic and functional frameworks for understanding conserved developmental processes relevant to rhizome biology. Representative references are cited in the main text

Rather than supplanting established rhizome systems, *Mimulus* (monkeyflowers) fills a distinct methodological gap by enabling rapid, iterative, and genetically clean tests of causality, and therefore represents one of the most promising complementary systems for advancing rhizome biology. Specifically, species in this genus possess traits absent from classical model systems such as *Arabidopsis*, including diverse floral pigmentation patterns, corolla tube formation, and robust rhizomatous growth (Fig. 2b) (Yuan 2019), and species within this genus share additional attributes that facilitate genetic and evolutionary research, including small genome sizes, short generation times, high fecundity, and extensive natural variation in mating systems, life-history traits, and edaphic preferences (Wu et al. 2008; Yuan 2019). Notably, at least four species complexes, namely, *M. aurantiacus*, *M. guttatus*, *M. luteus*, and *M. lewisii*, are amenable to *Agrobacterium*-mediated transformation (Yuan 2019), facilitating molecular genetic studies via transgenic perturbation. A side-by-side comparison of *Mimulus* with other model systems (Table 2) further underscores the unique suitability of *Mimulus* for advancing rhizome biology. This suitability has supported recent breakthroughs in the genetic control of floral pigmentation (Ding et al. 2020a; LaFountain et al. 2023; Sagawa et al. 2016; Stanley et al. 2020) and floral morphology (Chen et al. 2022; Ding et al. 2021, 2017, 2020b; Yuan et al. 2013a), based on approaches such as EMS mutagenesis and reverse genetics. Moreover, several causal genes underlying natural variation in flower color have been identified and functionally validated (Liang et al. 2023, 2022; Yuan et al. 2016, 2013b), advancing our understanding of the molecular mechanisms and evolutionary processes shaping phenotypic diversity in natural populations. Collectively, these attributes position *Mimulus* as a powerful and underutilized model system with exceptional potential for uncovering the genetic and developmental basis of rhizome traits.

Building on recent QTL mapping efforts in the *M.guttatus* species complex that revealed the genetic architecture of repeated life-history divergence in high-altitude perennials (Chen et al. 2025), future research should aim to dissect the genetic basis of rhizome variation in this system with greater precision. By integrating complementary approaches, including near-isogenic line development, fine-scale genetic mapping, multi-omics network reconstruction, and transgenic perturbation, it will be possible to identify key regulatory loci and decode the molecular mechanisms underlying rhizome initiation and elaboration. Such efforts will not only deepen understanding of the genetic and developmental mechanisms governing rhizome formation and growth, including how conserved regulatory modules may be differentially deployed across lineages, but will also establish *Mimulus* as a powerful and genetically tractable model for rhizome biology. Ultimately, this line of research has the potential to bridge a long-standing gap between developmental genetics and whole-plant functional biology, providing insights into how perennial plants persist across heterogeneous environments.

## Supplementary Information

Below is the link to the electronic supplementary material.

Supplementary file1 (DOCX 45 KB)

## Data Availability

No new datasets were generated or analyzed for this study.
