# Supplementary material for: The genetic and developmental enigma of rhizomes: crucial traits with limited understanding
Source: Theor Appl Genet. 2026 May 7;139(6):149. doi: 10.1007/s00122-026-05229-2 (PMC13153003; doi:10.1007/s00122-026-05229-2)
Supplement: Supplementary file 1 — Supplementary file1 (DOCX 45 KB) [file 122_2026_5229_MOESM1_ESM.docx]

**Table S1. Experimental conditions associated with reported hormonal or environmental effects on rhizome traits.** Opt-P, Optimal photoperiod; Opt-T, Optimal temperature; LD, long day; Low-T, low temperature. This table provides detailed experimental contexts corresponding to the qualitative effects summarized in Table 1.

| **Hormone / Factor** | **Species** | **System (in vivo / in vitro)** | **Tissue** | **Treatment (concentration / stress level)** | **Duration** | **Reported effect** | **Citation** |
| --- | --- | --- | --- | --- | --- | --- | --- |
| Auxin (IBA; NAA; IAA) | *Cymbidium forrestii* | in vitro | rhizome | 0.1–5 mg/L | 12 weeks | promotes rhizome initiation | Paek and Yeung 1991 |
| Auxin (NAA) | *Cymbidium goeringii* | in vitro | Apical flower buds | 1 mg/L | 8 weeks | promotes rhizome initiation | Shimasaki and Uemoto 1991 |
| Auxin (IAA) | *Leymus secalinus* | in vivo | whole plants | 0.005 mg/L | 14 days | promotes rhizome initiation | Li et al. 2022a |
| Auxin (NAA) plus Cytokinin (6-BA) | *Gastrodia elata* | in vitro | juvenile rhizome | 1–2 mg/L (NAA); 2 mg/L (BA) | 8 weeks | promotes rhizome elongation | Hsieh et al. 2022 |
| Auxin (IAA; IBA; NAA) | *Cymbidium aloifolium* | in vitro | rhizomes | 0.1–5 mg/L | 8 weeks | promotes rhizome branching | Nayak et al. 1998 |
| Auxin (NAA) | *Geodorum densiflorum* | in vitro | protocorm | 1 mg/L | 3 months | promotes rhizome branching | Roy and Banerjee 2002 |
| Auxin (IBA; NAA; IAA) | *Cymbidium forrestii* | in vitro | rhizome | 0.1–5 mg/L | 12 weeks | promotes rhizome branching | Paek and Yeung 1991 |
| Auxin (NAA) | *Geodorum densiflorum* | in vitro | protocorm | 0.5-4 mg/L | 2–6 months | promotes rhizome shooting | Roy and Banerjee 2002 |
| Auxin (NAA) | *Alstroemeria* | in vitro | rhizomes | 0.2 mg/L | 9 weeks | promotes rhizome shooting | Khaleghi et al. 2008 |
| Auxin (NAA) | *Alstroemeria* | in vitro | rhizome buds | 0.2 mg/L | 12 weeks | promotes rhizome shooting | Hamidoghli et al. 2007 |
| Auxin (IBA; IAA) | *Acorus calamus* | in vitro | rhizomes | 0.8 mg/L | 3 weeks | inhibits rhizome shooting | Tikendra et al. 2022 |
| Auxin (NAA) | *Ruppia maritima* | in vitro | terminal rhizome segments | 1 mg/L | 12 weeks | inhibits rhizome rooting | Koch and Durako 1991 |
| Auxin (NAA) | *Alstroemeria* | in vitro | rhizomes | 0.2 mg/L | 9 weeks | promotes rhizome rooting | Khaleghi et al. 2008 |
| Auxin (NAA) | *Alstroemeria* | in vitro | rhizome buds | 0.2–1 mg/L | 12 weeks | promotes rhizome rooting | Hamidoghli et al. 2007 |
| Auxin (NAA) | *Alstroemeria* | in vitro | rhizome | 1 µM | 7 weeks | promotes rhizome rooting | Kristiansen et al. 1999 |
| Auxin (IBA) | *Podophyllum hexandrum* | in vitro | rhizomes | 100 μM | 24 h | promotes rhizome rooting | Nadeem et al. 2000 |
| Auxin (NAA; IBA) | *Posidonia oceanica* | in vitro | rhizomes | 5 mg/L | 24 h | promotes rhizome rooting | Balestri and Lardicci 2006 |
| Auxin (IBA) | *Scopolia parviflora* | in vitro | rhizome | 2.46 μM | 4 weeks | promotes rhizome rooting | Kang et al. 2004 |
| Auxin (NAA) | *Valeriana jatamansi* | in vitro | rhizome | 1–1.5 mg/L | 8 weeks | promotes rhizome rooting | Nazir et al. 2022 |
| Cytokinin (6-BA) | *Cyperus serotinus* | in vitro | tubers | 0.1–10 mg/L | 14 days | promotes rhizome initiation | Omokawa et al. 1992 |
| Cytokinin (6-BA; kinetin) | *Kaempferia galang* | in vitro | rhizome | 6-BA: 13.2 µM; kinetin: 4.6 µM | 120 days | promotes rhizome shooting | Vincent et al. 1992 |
| Cytokinin (6-BA) | *Curcuma aromatica* | in vitro | shoots | 1–5 mg/L | 30 days | promotes rhizome initiation | Nayak 2000 |
| Cytokinin (6-BA) | *Zingiber officinale* | in vitro | shootlets | 6–9 mg/L | 10 weeks | promotes rhizome initiation | Abbas et al. 2014 |
| Cytokinin (6-BA; 2iP; Kinetin) | *Cymbidium forrestii* | in vitro | rhizome | 0.5–10 mg/L | 12 weeks | inhibits rhizome branching | Paek and Yeung 1991 |
| Cytokinin (Zeatin; 6-BA) | *Cymbiaium kanran* | in vitro | rhizome apical segments | 0.01–10 μM | 8 weeks | inhibits rhizome branching | Shimasaki 1995 |
| Cytokinin (6-BA) | *Geodorum densiflorum* | in vitro | protocorm | 0.5–8 mg/L | 3 months | promotes rhizome shooting | Roy and Banerjee 2002 |
| Cytokinin (6-BA) | *Geodorum densiflorum* | in vitro | protocorm | 0.5–8 mg/L | 3 months | inhibits rhizome rooting | Roy and Banerjee 2002 |
| Cytokinin (Kinetin; 6-BA; 2iP; Zeatin; thidiazuron) | *Ruppia maritima* | in vitro | terminal rhizome segments | 5–20 mg/L (BAP, 2iP, and zeatin); 10⁻⁹ (thidiazuron) | 12 weeks | inhibits rhizome rooting | Koch and Durako 1991 |
| Cytokinin (6-BA) | *Gastrodia elata* | in vitro | juvenile rhizome | 1 mg/L | 8 weeks | promotes rhizome elongation | Hsieh et al. 2022 |
| Cytokinin (6-BA; Kinetin) | *Cymbidium aloifolium* | in vitro | rhizomes | 0.25–1 mg/L | 8 weeks | promotes rhizome shooting | Nayak et al. 1998 |
| Cytokinin (Zeatin; 6-BA) | *Cymbiaium kanran* | in vitro | rhizome apical segments | 0.01–10 μM | 8 weeks | promotes rhizome shooting | Shimasaki 1995 |
| Cytokinin (6-BA) | *Alstroemeria* | in vitro | rhizomes | 0.5–2.5 mg/L | 9 weeks | promotes rhizome shooting | Khaleghi et al. 2008 |
| Cytokinin (6-BA) | *Cymbidium kanran* | in vitro | rhizome apex | 0.1–10 mg/L | 4 weeks | promotes rhizome shooting | Shimasaki and Uemoto 1990 |
| Cytokinin (6-BA; TDZ) | *Acorus calamus* | in vitro | rhizomes | 0.8–2.4 mg/L | 3 weeks | promotes rhizome shooting | Tikendra et al. 2022 |
| Cytokinin (6-BA、2iP) | *Alstroemeria* | in vitro | Rhizomes | 0.5–2 mg/L | 6–8 weeks | inhibits rhizome rooting | Gabryszewska and Hempel 1984 |
| Cytokinin (6-BA) | *Alstroemeria* | in vitro | rhizome | 10 µM | 7 weeks | inhibits rhizome rooting | Kristiansen et al. 1999 |
| Auxin (NAA) plus Cytokinin (6-BA) | *Cymbidium goeringii* | in vitro | Apical flower buds | 0.1 mg/L 6-BA; 10 mg/L NAA | 8 weeks | promotes rhizome initiation | Shimasaki and Uemoto 1991 |
| Auxin (NAA) plus Cytokinin (6-BA) | *Bambusa bambos* | in vitro | shoots | 2.5 µM 6-BA; 50 µM NAA | 3–4 weeks | promotes rhizome initiation | Kapoor and Rao 2006 |
| Auxin (NAA) plus Cytokinin (6-BA) | *Geodorum densiflorum* | in vitro | protocorm | 4 mg/L 6-BA; 1 mg/L NAA | 3 months | promotes rhizome initiation | Roy and Banerjee 2002 |
| Auxin (NAA) plus Cytokinin (6-BA) | *Geodorum densiflorum* | in vitro | protocorm | 0.5–4 mg/L 6-BA; 1 mg/L NAA | 3 months | Inhibits rhizome branching | Roy and Banerjee 2002 |
| Auxin (NAA) plus Cytokinin (zeatin) | *Zingiber officinale* | in vitro | shoots | 2.5 µM NAA; 10 µM zeatin | 14 weeks | promotes rhizome initiation | Zahid et al. 2021 |
| Auxin (NAA) plus Cytokinin (6-BA) | *Alstroemeria* | in vitro | rhizome tips | 1 mg/L 6-BA; 0.2 mg/L NAA | 12 weeks | promotes rhizome shooting | Shahriari et al. 2012 |
| Auxin (NAA) plus Cytokinin (6-BA) | *Valeriana jatamansi* | in vitro | rhizomes | 1–1.5 mg/L BAP; 0.5–1.5 mg/L NAA | 5–6 weeks | promotes rhizome rooting | Nazir et al. 2022 |
| gibberellic acid (GA₃) | *Bambusa bambos* | in vitro | shoots | 0.1 µM | 3–4 weeks | promotes rhizome initiation | Kapoor and Rao 2006 |
| gibberellic acid (GA₃) | potato | in vitro | etiolated sprout segments | 0.2–2 mg/L | 3 weeks | promotes rhizome initiation | Escalante and Langille 1995 |
| gibberellic acid (GA₃) | *Leymus secalinus* | in vivo | whole plants | 0.2 mg/L | 14 days | promotes rhizome initiation | Li et al. 2022a |
| gibberellic acid (GA₃) | *Chrysanthemum morifolium* | in vivo | whole plants | 100 μM | 50 days (every 10 days treatment) | promotes rhizome initiation | Zhang et al. 2022 |
| gibberellic acid (GA₃) | *Caulerpa prolifera* | in vitro | thalli | 1.67 μg/L | 14-25 days | promotes rhizome elongation | Jacobs and Davis 1983 |
| gibberellic acid (GA₃) | potato | in vitro | potato | 100 mg/L | 10 days | promotes rhizome elongation | Escalante and Langille 1998 |
| gibberellic acid (GA₃) | *Festuca arundinacea* | in vivo | whole plants | 10 μM | 12 days | promotes rhizome elongation | Ma and Huang 2016 |
| gibberellic acid (GA₃) | *Festuca arundinacea* | in vivo | whole plants | 10 μM | 12 days | promotes rhizome elongation | Ma et al. 2016 |
| gibberellic acid (GA₃) | *Cyperus esculentu* | in vivo | whole plants | 1000 ppm | 28 days | inhibits rhizome shooting | Garg et al. 1967 |
| gibberellic acid (GA₃) | *Rheum rhabarbarum* | in vitro | rhizome | 5 and 50 mg/L | 1 week | promotes rhizome shooting | Rayirath et al. 2009 |
| gibberellic acid (GA₃) | *Agropyron repens* | in vitro | rhizomes | 10 mg/L | 14 days | inhibits rhizome shooting | Rogan and Smith 1976 |
| gibberellic acid (GA₃) | *Zantedeschia* | in vitro | rhizome | 150 mg/L | 20 minutes | promotes rhizome shooting | Kozłowska et al. 2007 |
| gibberellic acid (GA₃) | *Convolvulus sepium* | in vitro | rhizome | 0.02–1 mg mg/L | 1 week | inhibits rhizome rooting | Wells and Riopel 1972 |
| Ethylene (Ethephon) | *Rheum rhabarbarum* | in vitro | shoot clumps | 1–50 mg/L | 6 weeks | promotes rhizome initiation | Rayirath et al. 2011 |
| Ethylene (Ethephon) | *Cymbidium kanran* | in vitro | shoots | 1–100mg/L | 7 weeks | promotes rhizome initiation | SHIMASAKI 1993 |
| Ethylene (Ethephon) | *Rheum rhabarbarum* | in vitro | shoot clumps | 1 mg/L | 6 weeks | promotes rhizome elongation | Rayirath et al. 2011 |
| Ethylene (Ethephon) | *Rheum rhabarbarum* | in vitro | shoot clumps | 1 mg/L | 6 weeks | promotes rhizome branching | Rayirath et al. 2011 |
| Ethylene (Ethephon) | *Cymbiaium kanran* | in vitro | rhizome apical segments | 10 μM | 8 weeks | promotes rhizome branching | Shimasaki 1995 |
| Ethylene (Ethephon) | *Cymbiaium kanran* | in vitro | rhizome apical segments | 10 μM | 8 weeks | inhibits rhizome shooting | Shimasaki 1995 |
| Ethylene (Ethephon) | *Cymbidium kanran* | in vitro | apical rhizomes | 10 mg/L | every 2 weeks, up to 4 months | inhibits rhizome shooting | Ogura-Tsujita and Okubo 2006 |
| Ethylene (Ethrel) | *Kohleria eriantha* | in vitro | rhizome | 10⁻³ M | 10 days | inhibits rhizome shooting | Almeida et al. 2005 |
| Ethylene (ethephon) | *Zingiber officinale* | in vitro | rhizomes | 750 ppm | 10 minutes | promotes rhizome shooting | Furutani et al. 1985 |
| Ethylene (Ethrel) | *Solanum tuberosum* | in vivo | whole plants | 10-100 ppm | 6 weeks | promotes rhizome elongation | Langille 1972 |
| Ethylene (ethephon) | *Solanum tuberosum* | in vio | whole plants | 50-1000 ppm | 6 weeks | promotes rhizome branching | Langille 1972 |
| Jasmonic acid | *Rheum rhabarbarum* | in vitro | shoot clumps | 10 ng/L–1 μg/L | 6 weeks | promotes rhizome initiation | Rayirath et al. 2011 |
| Abscisic acid | *Cnidium officinale* | in vitro | microshoots | 0.5 mg/L–1.0 mg/L | 8 weeks | promotes rhizome initiation | Kim et al. 2022 |
| Sucrose | *Bambusa bambos* | in vitro | shoots | 5% | 3–4 weeks | promotes rhizome initiation | Kapoor and Rao 2006 |
| Sucrose | *Kaempferia parviflora* | in vitro | rhizome buds | 6% | 9 weeks | promotes rhizome initiation | Labrooy et al. 2020 |
| Sucrose | *Zingiber officinale* | in vitro | shoots | 45 g/L–90 g/L | 12 weeks | promotes rhizome initiation | Zahid et al. 2021 |
| Sucrose | *Zingiber officinale* | in vitro | plantlets | 30–100 g/L | 12 weeks | promotes rhizome initiation | Gezahegn et al. 2024 |
| Sucrose | *Oryza longistaminata* | in vitro | Seedlings | 60–120 g/L | 3 months | promotes rhizome initiation | Fan et al. 2022 |
| Sucrose | *Bambusa bambos* | in vitro | shoots | 5% | 3–4 weeks | promotes rhizome elongation | Kapoor and Rao 2006 |
| Sucrose | *Acorus calamus* | in vitro | shoots | 2%–6% | 6 weeks | promotes rhizome elongation | Subramani et al. 2014 |
| Sucrose | *Oryza longistaminata* | in vitro | Seedlings | 60–100 g/L | 3 months | promotes rhizome elongation | Fan et al. 2022 |
| Sucrose | *Cymbidium forrestii* | in vitro | rhizome | 3–7% | 12 weeks | promotes rhizome shooting | Paek and Yeung 1991 |
| Sucrose | *Zingiber officinale* | in vitro | shoots | 45–60 g/L | 12 weeks | promotes rhizome shooting | Zahid et al. 2021 |
| Sucrose | *Zingiber officinale* | in vitro | shoots | 45–60 g/L | 12 weeks | promotes rhizome rooting | Zahid et al. 2021 |
| Sucrose | *Alstroemeria* | in vitro | rhizome | 20–60 g/L | 8 weeks | promotes rhizome rooting | Gabryszewska 1996 |
| Nitrogen | *Agropyron repens* | In vivo | Whole plants | 2.1 ppm | Transplanting → 6th leaf | promotes rhizome  initiation | McIntyre 1964 |
| Nitrogen | *Cyperus esculentus* | In vivo | Whole plants | Hoagland solutions of 1/32–1/2 | 28 days | promotes rhizome shooting | Garg et al. 1967 |
| Opt-P | *Curcuma aromatica* | in vitro | shoots | 8 h/day | 30 days | promotes rhizome initiation | Nayak 2000 |
| Opt-P | *Zingiber officinale* | in vitro | shoots | 8-24h/day | 8 weeks | promotes rhizome initiation | Rout et al. 2001 |
| Photoperiod | *Oryza longistaminata* | in vitro | rhizome | continuous light | 2 weeks | inhibits rhizome initiation | Yoshida et al. 2016 |
| Opt-P | *Cyperus rotundus* | in vitro | tubers | 25°C–35°C | 10 days | promotes rhizome initiation | Maria De Fatima and Valio 1976 |
| Opt-P | *Agropyron repens* | in vitro | rhizome | 13–23°C | 30 days | promotes rhizome shooting | Leakey et al. 1978 |
| LD | *Agropyron repens* | in vivo | Whole plants | 18 h | Transplanting → 6th leaf | promotes rhizome initiation | McIntyre 1967 |
| LD | *Caulerpa prolifera* | in vivo | whole plants | continuous light | 8 days | promotes rhizome elongation | CHEN 1971 |
| LD | *Trientalis boreal* | in vivo | wholeplants | 8–17h | 4 months | promotes rhizome elongation | Anderson 1970 |
| LD | *Poa pratensis* | in vivo | whole plants | - | 9 weeks | promotes rhizome elongation | Aamlid 1992 |
| LD | *Poa pratensis* | in vivo | whole plants | 16–18 h | 7–8 weeks | promotes rhizome elongation | Moser et al. 1968 |
| LD | *Nelumbo nucifera* | in vivo | whole plants | Summer long-day | 2 months | promotes rhizome branching | Masuda et al. 2006 |
| LD | *Alstroemeria* | in vivo | whole plants | 12–16 h | 2–11 weeks | inhibits rhizome branching | Vonk Noordegraaf 1981 |
| LD | *Cyperus esculentus* | in vivo | whole plants | 15½ h | 28 days | promotes rhizome shooting | Garg et al. 1967 |
| Low-T | *Agropyron repens* | in vivo | Whole plants | 10°C | Transplanting → 6th leaf | promotes rhizome initiation | McIntyre 1967 |
| Low-T | *Poa pratensis* | in vivo | Whole plants | 0–2 °C | 10–40 days | inhibits rhizome initiation | Moser et al. 1968 |
| Low-T | *Poa pratensis* | in vivo | whole plants | 0–2°C | 10–40 days | inhibits rhizome elongation | Moser et al. 1968 |
| Low-T | *Alstroemeria* | in vivo | whole plants | 9–13°C | 6–10 weeks | promotes rhizome branching | Vonk Noordegraaf 1981 |
| High-T | *Poa pratensis* | in vivo | whole plants | high day temperature (21 °C) | 9 weeks | promotes rhizome elongation | Aamlid 1992 |
| High-T | *Oryza longistaminata* | in vivo | whole plants | 28–30 ℃ | 3–4 weeks | promotes rhizome elongation | Wang et al. 2024a |
| High-T | *Nelumbo nucifera* | in vivo | whole plants | 20°C–30°C | 2 months | promotes rhizome branching | Masuda et al. 2006 |
| High-T | *Cyperus esculentus* | in vivo | whole plants | 33/27 ℃ (day/night) | 28 days | promotes rhizome shooting | Garg et al. 1967 |
| Drought | *Leymus chinensis* | in vivo | whole plants | 30%–35% field capacity | 90 days | inhibits rhizome initiation | Wang et al. 2019 |
| Drought | *Carex lasiocarpa* | in vivo | whole plants | Soil water content (49%, w/w) | 40 days | inhibits rhizome initiation | Yuan et al. 2017 |
| Drought | *Chrysanthemum morifolium* | in vivo | whole plants | relative water content (50%–10%) | 30 days | inhibits rhizome initiation | Zhang et al. 2022 |
| Drought | *Triglochin buchenaui* | in vivo | whole plants | 200 mL/2 weeks, bottom–watering | 3 months | promotes rhizome initiation | Tabot and Adams 2012 |
| Drought | *Leymus secalinus* | in vivo | whole plants | −50% growing-season precipitation | 6 years | promotes rhizome initiation | Zheng et al. 2021 |
| Drought | *Leymus chinensis* | in vivo | whole plants | 30%–35% field capacity | 90 days | inhibits rhizome elongation | Wang et al. 2019 |
| Drought | *Carex lasiocarpa* | in vivo | whole plants | Soil water content (49%, w/w) | 40 days | inhibits rhizome elongation | Yuan et al. 2017 |
| Drought | *Chrysanthemum morifolium* | in vivo | whole plants | relative water content (50%-10%) | 30 days | inhibits rhizome elongation | Zhang et al. 2022 |
| Drought | *Leymus secalinus* | in vivo | whole plants | −50% growing-season precipitation | 6 years | promotes rhizome elongation | Zheng et al. 2021 |
| Drought | *Kohleria eriantha* | in vitro | rhizome | 1 mL water (low moisture); PEG −6 MPa | 20 days | inhibits rhizome initiation | Almeida et al. 2005 |
| Drought | rhizoma perennial peanut | in vitro | rhizome | 159 mm (Mar–May) | 80 days | inhibits rhizome shooting | Rice et al. 1996 |
| Drought | *Phragmites australis* | in vivo | whole plants | 35–40% field capacity | 120 days | inhibits rhizome shooting | Mingyang et al. 2022 |
| Drought | *Phragmites australis* | in vivo | whole plants | 35–40% field capacity | 90 days | promotes rhizome shooting | Mingyang et al. 2022 |
| Drought | *Festuca arundinacea* | in vivo | whole plants | Soil volumetric water content: 6.3% | 7 days | inhibits rhizome initiation | Ma et al. 2020 |
| Drought | *Festuca arundinacea* | in vivo | whole plants | Soil volumetric water content: 6.3% | 7 days | inhibits rhizome elongation | Ma et al. 2020 |
